# PeakRanger: A cloud-enabled peak caller for ChIP-seq data

**DOI:** 10.1186/1471-2105-12-139

**Published:** 2011-05-09

**Authors:** Xin Feng, Robert Grossman, Lincoln Stein

**Affiliations:** 1 Department of Biomedical Engineering, Stony Brook University, Stony Brook, NY 11794, USA; 2 Cold Spring Harbor Laboratory, One Bungtown Road, Cold Spring Harbor, NY 11724, USA; 3 Ontario Institute for Cancer Research, MaRS Centre, South Tower, 101 College Street, Suite 800, Toronto, ON M5G 0A3, Canada; 4 Institute for Genomics & Systems Biology, The University of Chicago, Cummings Life Sciences Center 431A, 920 East 58th Street, Chicago, IL 60637, USA

## Abstract

**Background:**

Chromatin immunoprecipitation (ChIP), coupled with massively parallel short-read sequencing (seq) is used to probe chromatin dynamics. Although there are many algorithms to call peaks from ChIP-seq datasets, most are tuned either to handle punctate sites, such as transcriptional factor binding sites, or broad regions, such as histone modification marks; few can do both. Other algorithms are limited in their configurability, performance on large data sets, and ability to distinguish closely-spaced peaks.

**Results:**

In this paper, we introduce PeakRanger, a peak caller software package that works equally well on punctate and broad sites, can resolve closely-spaced peaks, has excellent performance, and is easily customized. In addition, PeakRanger can be run in a parallel cloud computing environment to obtain extremely high performance on very large data sets. We present a series of benchmarks to evaluate PeakRanger against 10 other peak callers, and demonstrate the performance of PeakRanger on both real and synthetic data sets. We also present real world usages of PeakRanger, including peak-calling in the modENCODE project.

**Conclusions:**

Compared to other peak callers tested, PeakRanger offers improved resolution in distinguishing extremely closely-spaced peaks. PeakRanger has above-average spatial accuracy in terms of identifying the precise location of binding events. PeakRanger also has excellent sensitivity and specificity in all benchmarks evaluated. In addition, PeakRanger offers significant improvements in run time when running on a single processor system, and very marked improvements when allowed to take advantage of the MapReduce parallel environment offered by a cloud computing resource. PeakRanger can be downloaded at the official site of modENCODE project: http://www.modencode.org/software/ranger/

## Background

The genome-wide characterization of chromatin protein binding sites and the profiling of patterns of histone modification marks is essential for understanding the dynamics of chromatin, unraveling the transcriptional regulatory code and probing epigenetic inheritance. The main technique for performing this characterization is chromatin immunoprecipitation (ChIP), coupled with massively parallel short-read sequencing (seq)[1-5]. Unlike its predecessor ChIP-chip [6,7], ChIP-seq provides improved dynamic range and spatial resolution[5].

After mapping sequenced ChIP reads to the reference genome, the first critical task of ChIP-seq data analysis is to accurately identify the target binding sites or regions enriched in histone marks [8]. Since downstream analysis relies heavily on the accurate identification of such binding sites or regions, a large number of algorithms have been proposed for peak calling[2,9-24].

**Figure 1:**
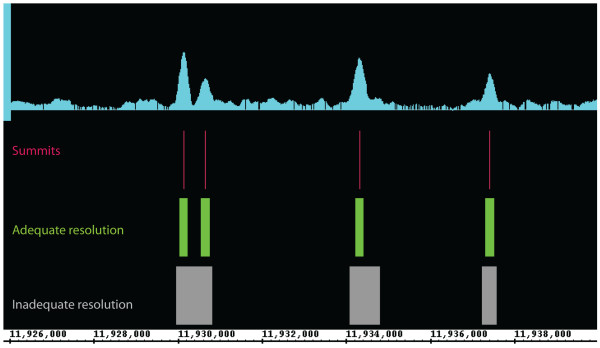
**The importance of peak caller resolution**. Some peak callers are designed to call surrounding enriched regions instead of summits. This degrades their ability to locate the site of binding events and their inter-peak resolution.

Despite the availability of such a large set of peak callers, many of these algorithms have disadvantages in real-world settings. Some algorithms have high sensitivity, but call an excessive number of false positive peaks due to low specificity. Others have the opposite problem. Another limitation of the current generation of peak callers is that many are optimized to detect either narrow punctate features, such as those generated by transcription-factor binding site experiments, or else optimized to detect broad peaks, such as those characterized by regions of modified histones. Hence a ChIP-seq production environment may need to install and maintain two different peak calling software packages. Those algorithms that attempt to handle both type of peak typically do so at the sacrifice of inter-peak and spatial resolution. The former is the ability to distinguish two or more closely-spaced peaks, while the latter is the ability to correctly locate the target binding site or histone modification boundaries. Both types of resolution are essential for understanding the underlying biology of chromatin dynamics. An example of how loss of resolution can affect the interpretation of ChIP-seq data is shown in Figure 1.

Software usability is also an issue. Some otherwise excellent peak callers are difficult to use because they require unusual data file formats, run slowly on real-world data sets, or do not take advantage of cluster computing. Poor usability can also impede the ability of a researcher to integrate the software with other tools in an analytic pipeline.

Here we present our efforts to address these concerns by creating PeakRanger, a novel peak caller that is both accurate and usable. Across a series of six accuracy benchmarks and three software usability benchmarks, it compares favorably to 10 other peak callers selected from the recent literature. In addition, PeakRanger supports MapReduce based parallel computing in a cloud environment, allowing it to scale well to large data sets in high-volume applications.

## Implementation

### Building the read coverage profile

The first step of peak calling is to build a read coverage profile using aligned raw reads. A key step in ChIP-seq is to shear the immunoprecipitated chromatin into fragments of 200-500 bp prior to extracting the DNA and sequencing it. Because the shear size is much larger than the small reads produced by early next-generation sequencing machines, many peak calling algorithms make use of the "shift" distance between coverage peaks defined by plus and minus strand read alignments, but this has become less useful as the read length produced by next-generation sequencers approaches the ChIP-seq DNA shear size. PeakRanger uses the same "blind-extension" strategy as PeakSeq[18] in which the shear size is provided by the user and not estimated from aligned raw reads. This choice significantly simplifies the software design and improves performance. (see additional file 1)

### Peak Detection

We first identify broad regions of signal enrichment using the same algorithm as PeakSeq, which detects contiguous enrichment regions by thresholding. After that, we use a "summit-valley-alternator" algorithm to scan for summits within regions identified by PeakSeq. This algorithm starts by searching for the first summit within the region, where a summit is defined as the location that has the maximum signal value before subsequent locations drop below a pre-defined cutoff value. The value is calculated by multiplying the current maximum signal value with delta, a tuning factor that should be chosen based on the needs of users. Delta is in the range (0, 1). Since the reads signal of broad regions are usually noisy, we perform additional signal processing before calling summits. (see additional file 1)

### Software Engineering

PeakRanger is written in C++, and can be compiled on Linux, MacOS and Windows. It runs as a command-line program.

## Results

### Benchmarking

In preparation for benchmarking, we compiled a list of 17 third-party peak callers mentioned in two recent reviews [8,25] plus several recently-published packages (see additional file 1). We attempted to install and run each peak caller on a test data set, and discarded seven that either failed to install, crashed during the test run, or produced no peaks from the test data set. This reduced the number of peak callers evaluated to 11, including PeakRanger.

#### Sensitivity benchmarks

**Figure 2:**
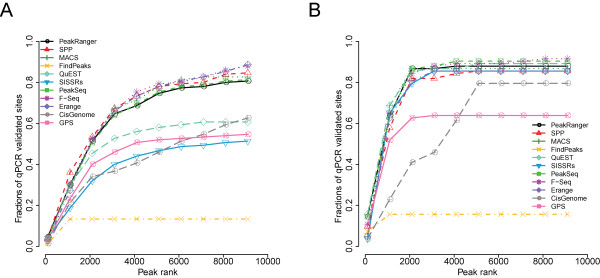
**Sensitivity test using qPCR validated ChIP-Seq binding sites**. The proportion of recovered qPCR validated binding sites is shown as a function of the ranked peaks called by each peak caller. Peaks are ranked based on significance values reported. A) Test results on the GABP dataset. B) Test results on the NRSF dataset.

In order to evaluate the sensitivities of the 11 algorithms, we evaluated them using two independent ChIP-seq datasets whose binding sites had been validated by qPCR[2,19]. Peaks called by each peak caller were ranked by their confidence scores and then compared to the list of validated sites. As measured by the average recovered proportion of validated sites, PeakRanger ranks within the top group, all of which have very similar sensitivities(Figure 2A).

#### Specificity benchmarks

It is more difficult to evaluate the specificity of peak calling than sensitivity because there is no golden standard of true-negative binding sites of sufficient size to confidently evaluate specificity. To partially address this issue, we performed a specificity analysis using a previously-published synthetic dataset [21]. This data set was generated from a real-world control (no antibody) experiment that contains no binding events, which was then spiked with simulated binding site peaks. Since all peaks were generated by the author, the locations of all simulated binding sites are known and false positive peaks can thus be defined.

**Figure 3:**
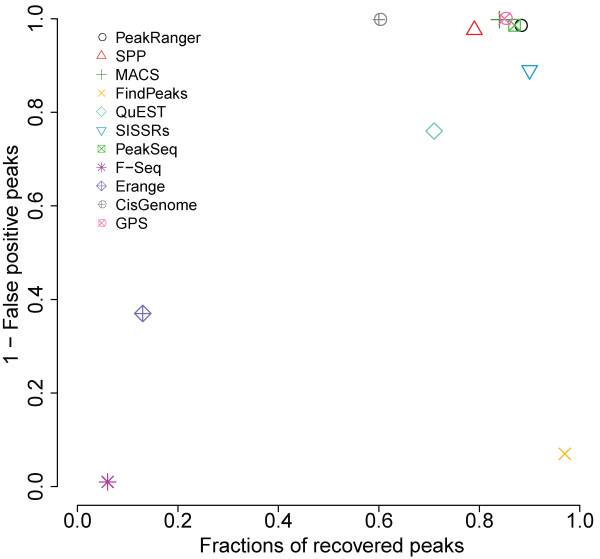
**The specificity test**. Peak calls of all peak callers on a semi-synthetic dataset are shown. All peak callers were configured to have a FDR cut off of 0.01. Recall rate is plotted against (1 - False positive rate)

Figure 3 graphs the true positive rate against (1-the false positive rate) for each of the peak callers at a fixed FDR rate of 0.01, as shown in Figure 3, in the top group, PeakRanger, PeakSeq, GPS and MACS have nearly the same good specificity and sensitivity. SPP is close to the top group. While SISSRs has higher sensitivity, it suffers from higher false positives. In contrast, although CisGenome called only a few false positive peaks, it recovered fewer peaks than the top group. F-Seq, Erange and FindPeaks all had unusually high false positive rates in this test.

#### Spatial accuracy benchmark

Spatial accuracy measures the ability of the peak caller to correctly identify the biological binding site underlying punctate peaks. To evaluate spatial accuracy, we again used the ChIP-seq data sets for the GABP and NSRF transcription factor targets. To identify the most likely biological binding sites, we used MAST[26] and the canonical target binding site motif and corresponding position specific scoring matrices (PSSMs) to find all matches in the 200 bp surrounding regions.

**Figure 4:**
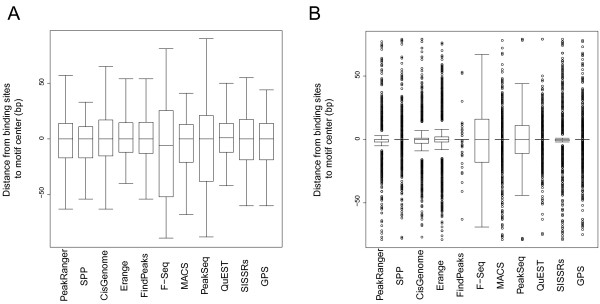
**The spatial accuracies of peak callers**. The distance from binding sites to motif center is measured for A) GABP and B) NRSF. Box-and-whisker plot is plotted to illustrate the distribution of distances from called binding sites to motif center for each peak caller.

We ran each of the peak callers on the data sets, and measured the distance between the binding site motifs and the centers of the closest overlapping peak call. As shown in Figure 4, algorithms that report peaks as single bp coordinates are much better than those that report broader regions. In particular, SPP, FindPeaks, GPS and QuEST were all tied for first place, closely followed by PeakRanger. However, the difference in spatial accuracy among the top-ranked peak callers is small.

#### Inter-peak resolution benchmark

This benchmark measures the ability of peak callers to distinguish between two closely-spaced peaks. This is a particularly difficult task for region-reporter algorithms, which tend to merge close peaks, potentially missing biologically-significant duplets. PeakRanger identifies closely-spaced summits within an enriched region by identifying local maxima within a smoothed model of coverage.

There are no real-world gold standard data sets for evaluating inter-peak resolution, so we adapted the semi-synthetic data set used previously for the specificity benchmarks. We created a series of derivative data sets to simulate closely spaced binding sites by generating a peak adjacent to each synthetic binding site. The inter-peak spacing varied from 200 to 500 bp in each of 13 derived data sets. To compensate for changes in coverage introduced by this modification, we added the same number of reads to the control. Some peak callers, including PeakRanger, provide a "resolution mode" that seeks to discover all summits within an enriched region. For this benchmark, we set each algorithm to use resolution mode or equivalent when available, or the default settings when not.

**Figure 5:**
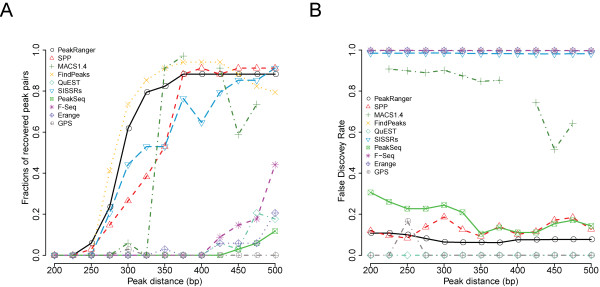
**Resolution test**. We called peaks on a series of semi-synthetic datasets consisting of paired peaks of increasing inter-peak separation. A) The percentage of close peaks recovered as the function of increasing inter-peak distance. B) The percentage of false positive peaks called. MACS crashed on the 200 bp, 400 bp and 500 bp datasets, so these data points are not plotted.

As shown in Figure 5A, no peak caller is able to resolve closely-spaced peaks in this data set when the peak separation is less than 250 bp. In the range of 250-350 bp, FindPeaks and PeakRanger lead the group in sensitivity, but FindPeaks produces an excessive number of false positives, as shown in Figure 5B. The other algorithms have lower sensitivities across this range and some exhibit very high false positive rates as well. MACS crashed on the 200 bp, 400 bp and 500 bp data sets, so these data points are missing.

### Usability design and performance tuning

Published algorithms are sometimes released in the research prototype stage, and do not have the software engineering necessary to work in a high volume, high availability setting. Ideally, a number of software engineering issues should be addressed (Table 1). First, the software should be as fast as possible. Our experience in large projects such as the modENCODE project[27] supports the notion that a faster peak caller will significantly reduce the time to analyze and interpret ChIP-seq data, because all the downstream analyses rely on accurate peak calls and there is often a cycle in which the results of downstream analyses inform additional rounds of peak calling using different parameter sets. Second, the software should support multiple common data formats. Transforming file formats requires extra time, computing resources, and introduces a step in which programming errors can creep in. Third, the software should be easy to use and requires less computing expertise from users. Finally, the software should be able to handle very large ChIP-seq data sets, given the rapid increase in next generation sequencing capacity.

**Table 1:** Usability summary of peak callers.

	GUI	Command line parameters input	Data format	Customizable input	Automatic format detection	Species	Reusable configuration file	Wiggle file generation	No preprocessing	Parallel processing	Cloud parallel computing
PeakRanger		Yes	Eland, Bowtie, SAM/BAM, BED	Yes		All	Yes	Yes	Yes	Yes	Yes

MACS		Yes	Eland, Bowtie, SAM/BAM, BED		Yes	All		Yes	Yes		

FindPeaks	Yes	Yes	Eland, Bowtie, BED, GFF			All		Yes			

SPP			Eland, Bowtie, MAQ, Arachne			All		Yes	Yes	Yes	

QuEST		Yes	Eland, Bowtie, Solexa, MAQ			All		Yes		Yes	

GPS		Yes	Eland, Bowtie, SAM, NovoAlign, BED			All			Yes		

Erange		Yes	Eland, Bowtie, Blat, BED			All		Yes			

CisGenome	Yes	Yes	Eland, BED			All		Yes			

F-Seq		Yes	BED			All		Yes	Yes		

SISSRs		Yes	BED			All			Yes		

PeakSeq			Eland			Human		Yes			

This table summarizes commonly supported software features by existing peak callers.

We implemented PeakRanger in the compiled C++ programming language to optimize performance. We avoided performance losses from disk I/O by keeping all working data in memory rather than in temporary files; this has the effect of trading a larger memory footprint for increased execution speed. To take advantage of modern multi-core processors, we also designed PeakRanger to use parallel processing.

To benchmark the performance of PeakRanger against other peak callers, we recorded the running time of them required to process a typical data set. As shown in Table 2, PeakRanger is more than twice as fast as the next fastest peak caller tested, while consuming an acceptable amount of memory.

**Table 2:** The performance of peak callers.

Algorithms	Elapsed time	Maximum memory footprint
PeakRanger	2m9s	2.9G
PeakSeq	5m11s	1.48G
SISSRs	15m18s	0.89G
FindPeaks	19m39s	4.2G
Erange	21m31s	0.81G
F-Seq	23m6s	7.27G
MACS	33m13s	1.04G
SPP	34m59s	1.98G
QuEST	36m51s	4.36G
CisGenome	55m39s	1.85G
GPS	64m18s	4.39G

Running time and memory footprint was recorded for peak callers using the GABP dataset.

To enable the support of multiple input data formats, we adopted designs shared by SPP and MACS which separate data loading from data processing. We wrote individual modules for specific data formats and let users to choose the one they need. PeakRanger currently supports Bowtie[28], Eland, SAM[29] and BAM[29] formats. Other file formats can be added by writing additional importation modules. PeakRanger is also capable of exporting its results in formats suitable for data visualization, including both compressed and uncompressed versions of the UCSC Genome Browser "wiggle" format.

To support multiple species, peak calling packages need basic genome build information such as the names and sizes of chromosomes. For users' convenience, PeakRanger can either derive this information directly from the input files, or can be given pre-computed genome tables. Although the former mode is convenient, it does add a small amount of overhead to the execution time.

Although hard to quantify, we noted considerably variation in the difficulty of installing and configuring the various peak caller packages during our benchmarking tests. For example, some packages require the user to make changes to the source code in order to change the location of hard-coded file paths and run-time parameters. PeakRanger makes all its run-time configuration parameters available as command-line options, and also provides a reasonable set of presets for common analysis tasks. For example, PeakRanger provides "resolution mode" and "region mode", which are presets suitable for analyzing transcription factor binding sites and other punctate data on the one hand, and broad regions such as histone modifications on the other. All run-time parameters can be read from external configuration files as well, allowing parameter sets to be managed by source code control, versioned, and shared among laboratories.

PeakRanger does not provide a graphical user interface (GUI) such as those provided by CisGenome, USeq and Sole-Search[10]. While GUIs are convenient for casual users, they make it difficult to integrate the software into the automatic workflows needed by high-throughput laboratories, which are the target audience for PeakRanger.

### Support for MapReduce

With sequencing industry's rapidly increasing capacity to generate more and longer sequencing reads[30], peak calling algorithms face an exponentially growing demand for computational resources. Cloud computing[31] offers a cost-effective solution for groups that have highly variable demands for compute resources.

Current cloud computing infrastructures offer a highly scalable parallel computational model called MapReduce[32] which was originally designed by Google to process very large-volume datasets. We thus also implemented a MapReduce version of PeakRanger on top of the Hadoop library[33], a free open source implementation of MapReduce.

The Hadoop version of PeakRanger supports splitting the job by chromosomes to take advantage of the chromosome-level independence (CLI) of ChIP-seq data sets. Other ways of partitioning the genome are possible, but require additional preparation by the user.

**Figure 6:**
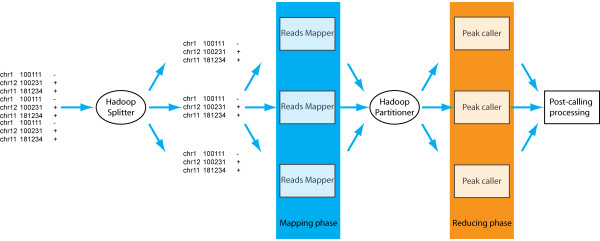
**The programming model of Hadoop and the adaptation of PeakRanger to it**. Reads are first splitted by the Hadoop spliter. Mappers are then initiated to preprocess these reads by chromosomes. Hadoop partitioner then assign processed reads to individual reducers to call peaks. Called peaks then undergo post-call processing.

Within the Hadoop framework, a PeakRanger job can be expressed as a series of "map-then-reduce" sub-jobs (Figure 6). PeakRanger first starts a series of mappers to map the input datasets to a set of keys. Then a Hadoop partitioner assigns keys to a set of reducers. Each individual reducer fetches the data according to the keys it receives and processes these data. In the CLI case, "map-then-reduce" becomes "split-by-chromosome-then-call-peaks" where chromosomes are used as keys. That is, we delegate the data loading/preprocessing to mappers and peak calling to reducers. After mappers finishes splitting data on chromosome, the partitioner assigns jobs based on the number of available reducers and reducers then do the actual peak calling.

To evaluate the performance of Hadoop-PeakRanger, we performed two benchmark tests: 1) test with fixed number of nodes and data sets of increasing size; 2) test with increasing numbers of nodes and data sets with fixed sizes.

**Figure 7:**
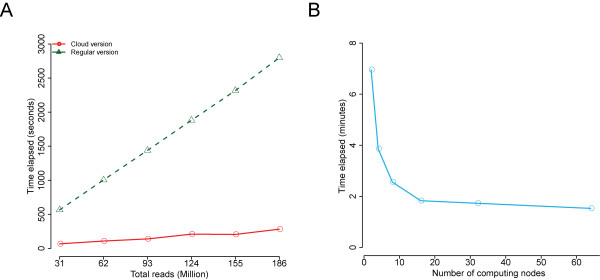
**Performance of PeakRanger in cloud parallel computing**. A) test with fixed number of nodes and data sets of increasing size; B) test with increasing numbers of nodes and data sets with fixed sizes.

Figure 7A demonstrates that on a fixed number of nodes with increasing data set sizes, the execution time for the Hadoop version of PeakRanger is dramatically shorter, and increases more slowly, than the regular single-processor version. For example, the cloud version processed 14 Gb dataset of 192 million reads in less than 5 minutes, more than 10 times faster than the original PeakRanger.

In the second test, we tested how the running time scales with the increasing number of nodes (Figure 7B). As expected, runtime decreases rapidly until the number of nodes equals the number of chromosomes (25), after which adding additional nodes does not provide further benefit. Future versions of PeakRanger will provide alternate ways of splitting the genome to overcome this parallelization bottleneck.

We plan to make both the regular and Hadoop version of PeakRanger available as public machine images in Amazon EC2 and other cloud service providers in order to facilitate its use by the research community.

### Real world usage of PeakRanger

In this section we provide two examples of using PeakRanger in biological research settings.

#### Characterization of broad enriched regions

It is common for studies of histone modifications to identify broad regions enriched in the modification of interest and then to correlate these broad regions with other biological annotations such as genes. Although this type of analysis is straightforward, it ignores the detailed internal structure of the enriched profiles, which can contain summits and valleys relating to quantitative differences in modification efficiency and/or heterogeneity within the sample.

Recently there have been several publications reporting biologically significant phenomena based on the internal structures of the enriched histone modification regions [34-36]. Therefore it is desirable that a peak caller be able to retrieve both broad enriched regions while simultaneously identifying the detailed summits within these regions. Here we demonstrate such an example using PeakRanger.

In the paper recently published by He et al[34], the authors found that after exposures to 5-α-dihydrotestosterone (DHT) the central nucleosome was depleted from a subpopulation of androgen receptor (AR) binding sites, leaving a pair of flanking nucleosomes. Without knowing the region structure in advance, it is difficult to identify the paired nucleosomes from the read coverage signal alone, and He et al built additional models to identify and quantify the paired binding sites.

**Figure 8:**
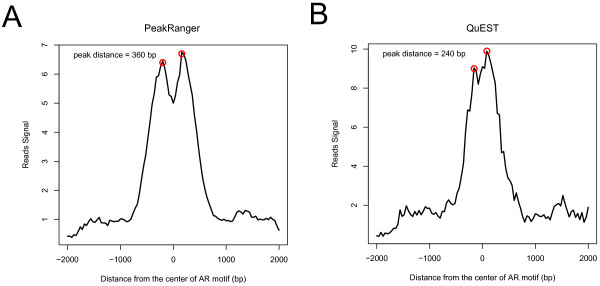
**Estimating the peak distance from DHT sensitive subgroups**. The analysis conducted by He et al is repeated by using just peak calls generated by A) PeakRanger and B) QuEST. PeakRanger gave a much closer estimate of the twin-peak distance than QuEST.

We applied PeakRanger directly to the He data set, using a configuration that allowed it to find both broad enriched regions and summits within the regions. We then compared the number of summits in each enriched region before and after DHT exposure to directly identify the subpopulation of AR binding sites that have depleted central nucleosomes. In order to accomplish this objective, we configured PeakRanger to detect summits with comparable heights. As shown in Figure 8A, the profile plot strongly resembled that reported in the original publication, and had an average twin-peak separation of 360 bp, close to the publication estimate of 370 bp. As a comparison, we repeated the same procedure using QuEST. The resulting estimated peak distance was 240 bp and the profile plot departed from the original one(Figure 8B). For other peak callers, since no information is available for the number of summits of an enriched region, we could not perform the same analysis.

#### Processing modENCODE worm datasets

ModENCODE is a multi-center collaboration to catalogue functional elements in C. elegans and D. melanogaster [27,37], and includes more than 100 ChIP-seq data sets. PeakRanger was used by modENCODE as the standard ChIP-seq peak caller for 29 ChIP-seq experiments for involving 23 C. elegans transcription factors across various developmental stages[37]. PeakRanger was able to process the entire data sets in less than 2 hours running on a regular workstation with 8G ram and a quad core CPU. This illustrates PeakRanger's ability to integrate into a high-throughput environment. Ultra-high through-put enabled great collaborated analysis among different labs. A couple of internal analysis shows that peaks produced by PeakRanger were of high quality (Data not shown).

## Discussion

**Figure 9:**
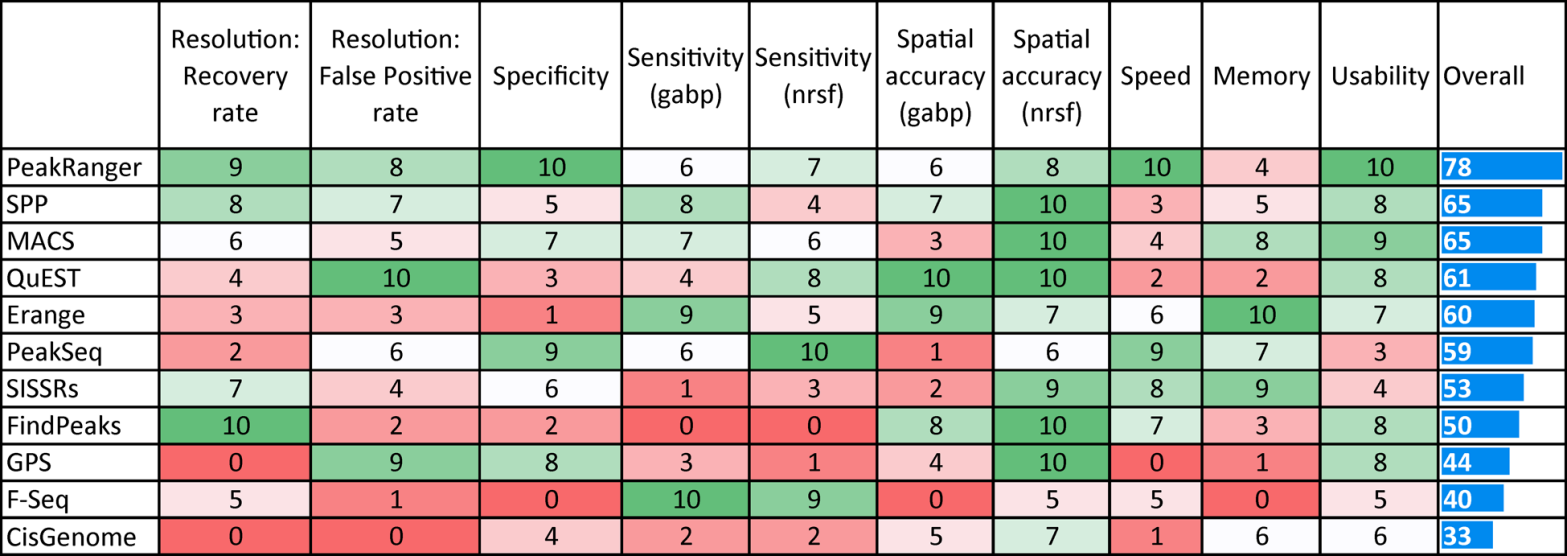
**Summary of benchmarks performed in this study**. For each benchmark item, peak callers are ranked and scored (see methods). The score has a range of 0 to 10 and 10 is the best score. The overall rank is based on the sum of all scores in all benchmarks.

Figure 9 summarizes the accuracy and software engineering benchmarks discussed above, where each of the 11 peak callers examined is ranked from 0 (worst) to 10 (best) for a particular benchmark. The last column of the table is a simple sum of the ranks. No single peak caller ranks as the best on all benchmarks; in particular, algorithms with high sensitivity often have low specificity. However, PeakRanger manages a good compromise among all the performance benchmarks and ranks first in the aggregate ranking.

The algorithm used to find the summits within enriched regions are similar to those used by QuEST and FindPeaks. To make the summit detection more reliable and flexible, we enhanced it based on our experiences of real ChIP-Seq datasets. In QuEST, users can not control the sensitivity of summits detection. In comparison, PeakRanger allows users to specify the sensitivity by using the -r option. We also applied an additional padding algorithm to avoid calling false positive summits. In case a dataset does not have adequate sequencing depth, we pad enriched regions so that the summit detection algorithm will not call summits if two base pairs are separated with regions of zero read counts.

PeakRanger relies on PeakSeq which detects enriched regions before the step of summit detecting. PeakSeq is an effective algorithm but the original implementation gives only limited usage of the algorithm. We thus significantly modified PeakSeq so that it can be integrated as a part of PeakRanger. PeakSeq contains two separate parts: pre-processing and peak-calling. These two parts are now combined into a single module to reduce file I/O cost. We also designed indexing of chromosomes to enable support to other species with different number and names of chromosomes. The original PeakSeq runs in single-thread mode and we modified related data structures to support multi-thread mode.

Although PeakRanger represents a successful compromise among multiple measures of accuracy, researchers should consider one of the other peak calling algorithms if a particular performance characteristic is of the top priority. For example, if identifying the precise center of the peak is critical to an experiment, then researchers should consider GPS, QuEST, MACS, SPP or FindPeaks, all of which have better spatial accuracy than PeakRanger.

The current design for the Hadoop version is based on chromosome-level-independence (CLI), which limits the practical level of parallelization to the number of chromosomes in the genome. This concept can be generalized to region-level-independence (RLI) by breaking the genomes into a set of arbitrary regions and call peaks in each individual region. However, this is dependent on the peak calls for each region being independent of each other, a criterion that is not satisfied when an enriched region crosses the region boundary. Additional manipulation of the regions to allow for overlap between them, and adjustments for the changes in coverage in overlapped regions will be necessary to implement this, and is deferred to future work. However, even with the current design we are able to archive an order-of-magnitude increase in speed, which is sufficient for most practical applications.

## Conclusion

In this paper, we introduce PeakRanger, a general purpose ChIP-seq peak calling algorithm that is optimized for accuracy, speed and ease of use. It is suitable for use in small laboratories, as well as in large production centers, and can be used in a cloud environment for very high throughput environments. The software is freely available and open source under the Artistic License 2.0. The primary download site is http://www.modencode.org/software/ranger/.

## Availability and requirements

PeakRanger is under the Artistic License 2.0. PeakRanger can be downloaded from: http://www.modencode.org/software/ranger/. We currently provide the full source code, as well as binaries for Linux systems. Binaries for other operating system and an Amazon EC2 image will be available during the first quarter of 2011.

## Authors' contributions

XF designed, implemented and tested the algorithm. LS helped testing the algorithm. RG provided hardware and software support for the cloud-enabled version. XF and LS wrote the manuscript. All authors read and approved the final manuscript.

## Supplementary Material

Additional file 1 **This file contains detailed description of the algorithms and benchmarks**. Click here for file
