# Supplementary material for: PeakRanger: A cloud-enabled peak caller for ChIP-seq data
Source: BMC Bioinformatics. 2011 May 9;12:139. doi: 10.1186/1471-2105-12-139 (PMC3103446; doi:10.1186/1471-2105-12-139)
Supplement: Additional file 1 — This file contains detailed description of the algorithms and benchmarks. [file 1471-2105-12-139-S1.PDF]

Methods

Read coverage profile building and detection of the enriched regions

The procedures for building reads profiles and peak calling are based on those used by PeakSeq[1] with the following modifications: Application of a mappability map is removed to enable support for multiple species. Similar with PeakSeq, PeakRanger uses the "blind-extension" algorithm to extend each read artificially to match the size of the shared DNA. The reads on the negative strand is also extended using the formula: (mapped_read = original_read - extension_length + read_length). The extension_length by default is set to 200 and can be adjusted using the -l option. Reads are then summed up to generate the read coverage profile. The algorithm of detection of enriched regions is the same one used by PeakSeq.

Coverage profile enhancement and summit detection

For each enriched region identified, we scan for summits in it using the coverage profiles used for region detection. The read coverage profile is padded prior to summit detection. The original profile is scanned and locations with zero read counts are detected. These locations are padded with the average value of the two nearest non-zero coverage regions. The regions are then smoothed using moving average algorithm. The window size for smoothing can be tuned using the -b option. Our tests show that the window size for smoothing should be no larger than half of the read extension length(data not shown).The padded profiles are then scanned for summits. The summit-valley-alternator algorithm starts by searching for the coordinate with reads maxima in the region. Then, all the remaining coordinates that have above-threshold reads are selected as summits. The threshold is obtained by multiplying the region-maximum value with a tuning factor (Delta) in the range (0, 1). Smaller Delta results in more summits and vice. versa. By controlling the -r option, users have flexible control of the sensitivity. The figure below shows some examples of how the summit detection algorithm works.

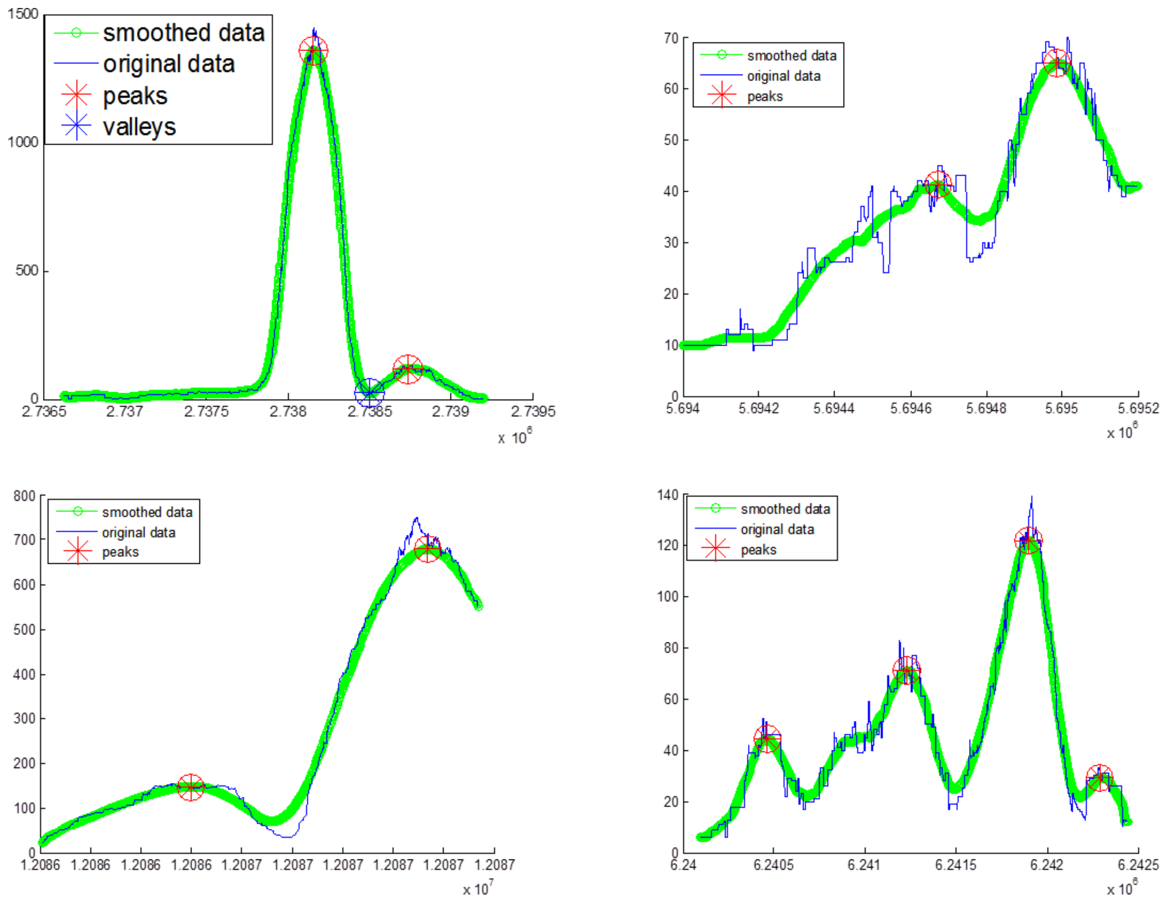

Algorithm implementation

PeakRanger is implemented using C++ and is open source. It compiles and runs on any operating systems that support the GNU G++ development environment. PeakRanger includes source files from PeakSeq, Bowtie and Bamtools[2]. Valgrind[3] tests for possible memory leaks are done for all tests described in this manuscript. Additional Valgrind tests were done using private datasets. The support for cloud computing relies on the Hadoop library[4].

Selection and configuration of peak callers

We based our selection of peak callers on two recent reviews [5, 6] to represent the algorithm diversity and popularity. We also added recently-published algorithms which had not been included in the reviews. This resulted in an initial set of 17 candidate peak callers(shown in table below), which we then screened to exclude callers that could not be compiled, required additional data files that we could not provide, or failed to produce peak calls in an initial test set. After screening, we finally included 10 peak callers in remained. All programs were run with their default/recommended settings. Tests were done in a generic desktop with the following specs: CPU: Intel Q6600, RAM: 12G, Harddisk: 2TB 7200 rpm.

Algorithms	Reference	Version	Initial Screening	Notes
ERANGE	23	3.2.1	PASSED	
FindPeaks	14	4	PASSED	
F-Seq	15	1.84	PASSED	
GLITR	16	literature version	FAILED	Requires too many control tags, More than 4X the total number of treatment tags are required
MACS	17	1.3.7.1 and 1.4.0beta	PASSED	1.4.0beta was used for the resolution test
PeakSeq	18	1.01	PASSED	The package programmed using the C programming language was used
QuEST	19	2.4	PASSED	
SICER	13	1.03	FAILED	Would not run without significant modifications
SISSRs	12	1.4	PASSED	
SPP	20	1.8	PASSED	
Useq	21	7.0	FAILED	Obtained zero peaks from test datasets.
Minimal ChipSeq Peak Finder	2	literature version	FAILED	Release webpage is missing
CisGenome	11	1.2	PASSED	Only Linux core programs were used
HPeak	24	2.1	FAILED	Program reported missing files during installation
Sole-Search	10	1.0	FAILED	Command line version is not available
CSDeconv	9	literature version	FAILED	Took more than 1 day to complete initial test set
GPS	22	0.10.1	PASSED	

Sensitivity test

The GABP dataset and NRSF dataset were downloaded from the website of QuEST (http://mendel.stanford.edu/SidowLab/downloads/quest/). The qPCR validation list was downloaded from [5]. Peaks were ranked based on the metrics provided by each peak caller. For F-Seq, which identified too many peaks, only the top 10,000 ranked peaks were used.

Specificity test

The original dataset used in the resolution test was from the website of USeq (http://sourceforge.net/projects/useq/). Peak callers were configured to have FDR 0.01 when calling peaks.

Spatial accuracy test

The GABP dataset and NRSF dataset were downloaded from the website of QuEST (http://mendel.stanford.edu/SidowLab/downloads/quest/). PSSMs were obtained from TRANSFAC[7]. The MAST[8] program from the MEME software suite was used to detect motif occurrences[9]. Boxplots were generated with R[10]. Only peaks within 100bp of a motif are retained for calculation.

Resolution test

The original dataset used in the resolution test was from the website of USeq(http://sourceforge.net/projects/useq/). Peaks were systematically shifted and reintroduced into the dataset to produce a series of synthetic peak pair datasets. We excluded CisGenome from the test because it failed to complete the benchmark. MACS version 1.4.0 beta was used in this test instead of MACS 1.3.7.1 since the latter does not have the ability to call multiple summits within a region. For the PeakRanger benchmark, we used a delta value of 0.2 to enable the ability to call multiple summits. For QuEST, we used a dip_fraction of 0.8 because QuEST uses a threshold value of (1-dip_fraction) X (maxima reads). For FindPeaks, we used a -subpeaks option of 0.2 for the "-subpeaks" option. We calculated recovery rate and false discovery rate using custom Java programs.

Histone modification usage example

The dataset was downloaded from GEO using the accession ID: GSE20042. We used a delta value of 0.4 for PeakRanger, and a dip_fraction of 0.6 for QuEST.

Speed and memory footprint test

We used the GABP dataset. SPP gave us an error message when we attempted to run it with parallel support, so it was run in the regular non-parallel mode. We ran PeakRanger with the "-t 4" option to enable parallel processing. QuEST automatically launched multiple processing sub-programs. All other peak callers were run in regular non-parallel modes. All peak callers were tested in the same computer with 12G memory and a quad-core CPU.

Testing the Hadoop-PeakRanger

We chose Eucalyptus as the cloud controller. We wrote scripts to deploy Hadoop across a set of allocated cloud nodes, and utility scripts to start, stop and checking the Hadoop server. We built cloud executables using a virtual system image based on Debian Linux, and then populated with the Hadoop binaries, PeakRanger and its

support files. Execution of the Hadoop version of PeakRanger uses the Hadoop Streaming system.

Plots and data visualizing
Signal tracks of Figure 1 and is drawn using the IGB browser[11].

Preparation of Summary Table
For the summary table Figure 9, we ranked each peak caller based on its relative performance in each benchmark. For the resolution:recovery test, we ranked average recovery rate. For the resolution:false discovery rate, we ranked average false discovery rate. For the specificity test, we ranked recovery rate minus false discovery rate. For the spatial accuracy test, we ranked the absolute distance between the higher and lower hinge of the distance distribution. For the sensitivity test, we ranked the average recovery rate. For the speed test, we ranked elapsed clock time. For the memory test, we ranked the peak memory footprint consumed during execution. For the usability test, we ranked the sum of the features listed in Table 2.

Command line parameters and sample usages
PeakRanger requires a treatment and a control file to run. Users can specify these two files with -d and -c options. The format of the files must also be specified using the --format option. Additionally, users should specify the output location for the result files. An example below shows the basic usage:

ranger --format=fileformat -d treatment -c control -o outputlocation

Other parameters may also be useful and are documented in the manual included in the software package. For example, the number of threads can be specified with -t; To specify the FDR cut-off, use -p; To show the overall processing progress, use --verbose.

References

1. Rozowsky J, Euskirchen G, Auerbach R, Zhang Z, Gibson T, Bjornson R, Carriero N, Snyder M, Gerstein M: **PeakSeq enables systematic scoring of ChIP-seq experiments relative to controls**. *Nat Biotechnol* 2009, **27**:66 - 75.
2. **Bamtools** [http://github.com/pezmaster31/bamtools]
3. **Valgrind** [http://valgrind.org/]
4. **Hadoop** [http://hadoop.apache.org/]

5. Wilbanks EG, Facciotti MT: **Evaluation of Algorithm Performance in ChIP-Seq Peak Detection**. *PLoS ONE* 2010, **5**(7):e11471.
6. Pepke S, Wold B, Mortazavi A: **Computation for ChIP-seq and RNA-seq studies**. *Nat Meth* 2009, **6**(11s):S22-S32.
7. Wingender E, Dietze P, Karas H, Knüppel R: **TRANSFAC: A Database on Transcription Factors and Their DNA Binding Sites**. In., vol. 24; 1996: 238-241.
8. Bailey TL, Gribskov M: **Combining evidence using p-values: application to sequence homology searches**. *Bioinformatics* 1998, **14**(1):48-54.
9. Bailey TL, Elkan C: **Fitting a mixture model by expectation maximization to discover motifs in biopolymers**. *Proc Int Conf Intell Syst Mol Biol* 1994, **2**:28-36.
10. Team RDC: **R: A Language and Environment for Statistical Computing**; 2008.
11. Nicol JW, Helt GA, Blanchard SG, Raja A, Loraine AE: **The Integrated Genome Browser: free software for distribution and exploration of genome-scale datasets**. In., vol. 25; 2009: 2730-2731.
